# Rapid and Non-Destructive Repair of Fused Silica with Cluster Damage by Magnetorheological Removing Method

**DOI:** 10.3390/mi12030274

**Published:** 2021-03-06

**Authors:** Mingjie Deng, Ci Song, Feng Shi, Yaofei Zhang, Ye Tian, Wanli Zhang

**Affiliations:** 1 College of Intelligence Science and Technology, National University of Defense Technology, 109 Deya Road, Changsha 410073, China; dengmingjie19@163.com (M.D.); sf.wind@yahoo.com (F.S.); zhangyaofei18@nudt.edu.cn (Y.Z.); tianyecomeon@sina.cn (Y.T.); zhangwanli17@nudt.edu.cn (W.Z.); 2 Hunan Key Laboratory of Ultra-Precision Machining Technology, Changsha 410073, China; 3 Laboratory of Science and Technology on Integrated Logistics Support, National University of Defense Technology, 109 Deya Road, Changsha 410073, China

**Keywords:** fused silica, small-scale damage, magnetorheological removing method, combined repairing process, evolution law

## Abstract

The damage repair of fused silica based on the CO_2_ laser repair technique has been successfully applied in high-power laser systems in the controllable nuclear fusion field. However, this kind of repairing technique mainly focuses on large-scale laser damage with sizes larger than 200 μm, but ignores the influence of cluster small-scale damage with sizes smaller than 50 μm. In order to inhibit the growth of small-scale damage and further improve the effect of fused silica damage repair, this paper carried out a study on the repair of fused silica damage using the magnetorheological (MR) removing method. The feasibility of fused silica damage repairing was verified, and the evolution law of the number, morphology, and the surface roughness of small-scale damage were all analyzed. The results showed that the MR removing method was non-destructive compared to traditional repairing technologies. It not only effectively improved the whole damage repairing rate to more than 90%, but it also restored the optical properties and surface roughness of the damaged components in the repairing process. Based on the study of the MR removing repair law, a combined repairing process of 4 μm MR removal and 700 nm computer controlled optical surfacing (CCOS) removal is proposed. A typical fused silica element was experimentally repaired to verify the process parameters. The repairing rate of small-scale damage was up to 90.4%, and the surface roughness was restored to the level before repairing. The experimental results validate the effectiveness and feasibility of the combined repairing process. This work provides an effective method for the small-scale damage repairing of fused silica components.

## 1. Introduction

Due to its excellent optical properties, fused silica is often used as a diffractive optical element and a focusing optical lens in the terminal device of high-power laser systems [1,2]. As high-powered laser optics are difficult to fabricate and easy to damage during application, it is very difficult to meet the requirements only by manufacturing methods [3]. Therefore, methods of repairing and reusing damaged components are urgently needed [4].

**Figure 1:**
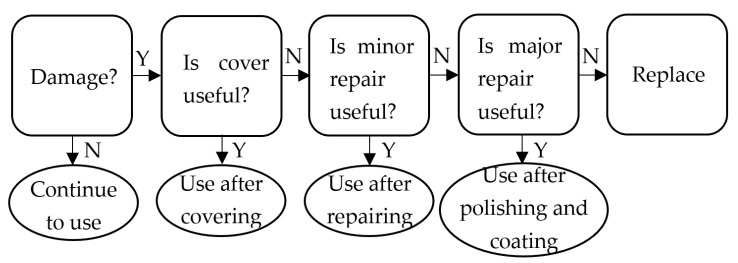
Flow chart of optical elements in the NIF system.

Figure 1 shows the flow chart of the optical elements in the National Ignition Facility Project (NIF) system [5]. Three processing methods are proposed for damaged elements: firstly, the damaged area is covered; secondly, if it cannot be covered, it needs to be repaired; thirdly, if it cannot be repaired, it should be replaced [6]. As an important part of the component in the NIF system, damage repair has become key to ensure the stable operation of the whole system.

At present, the surface damage repairing methods of fused silica components include HF etching, femtosecond laser repairing, and CO_2_ laser repairing, among others. CO_2_ laser repairing is the most effective and reliable method and has been widely studied and developed [7,8,9,10]. Although CO_2_ evaporation repairing has been used in engineering and has a good inhibition effect on damage growth, there are still some problems in practical application [11,12]. This method is mainly used to repair single point damage where sizes are larger than 200 μm, and the optical performance of the repairing point will be invalid after repairing [11,13]. A large number of small-scale damage points are difficult to repair with this method because the cost and time of repair are greatly increased [14].

**Figure 2:**
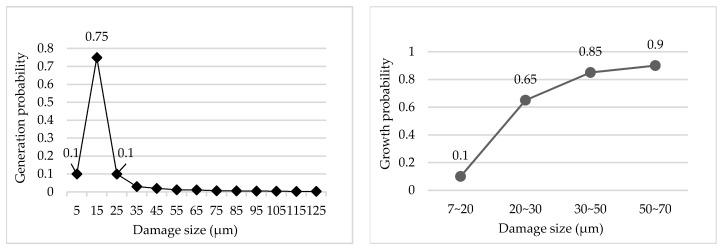
Probability of damage generation and growth.

Miller from the Lawrence Livermore National Laboratory, California, USA conducted an in-depth study on the law of damage occurrence and growth, as shown in Figure 2. The research showed that the probability of small-scale damage points with sizes less than 50 μm was more than 95%, and the probability of growth was very high. That is to say, a large number of small-scale damage points would lead to rapid deterioration, and ultimately affect the service life of components [15,16]. Miller used a new terminal optical component damage detection system called the Final Optics Damage Inspection (FODI), which can reliably detect damage sites larger than 50 microns in the repairing process. Then, the small damage points can be repaired and the service life of components can be improved by 40% [15]. Therefore, to the ability to repair small damage points and inhibit their further growth quickly and effectively is critical to improving the optical performance of fused silica optical elements [17].

In order to achieve batch and high-efficiency removal of the small-scale damage points without inducing new damage, this paper adopts the magnetorheological (MR) removing method to repair the damaged fused silica components. The MR removing method has the characteristics of high-efficiency removal, strong controllability, and good surface quality [18]. The biggest difference between the MR removing and traditional methods is that it is based on the shear removal. The process of repairing the original damage points of components mainly occurs in the elastic-plastic domain and does not induce new crushing defects [19].

Guo Zhongda of Northwestern Polytechnic University, Xi’an, China found that if the surface of the optical element is uneven and the magnetic field intensity in the depression is positioned where the gradient magnetic field is relatively weak, the magnetic particles will move to the depression and reduce the extrusion of the optical parts, thus forming comet tail defects and degrading the surface quality [20].

Zhang Yaofei of the National University of Defense Technology, Changsha, China proposed a method of repairing cluster small-scale damage by using the MR removing method, and carried out a preliminary study on the evolution law of MR repairing damages on fused silica [21]. The results showed that MR repair produced a tail and worsened the surface quality. Follow-up research was carried out, and the feasibility of MR repairing damage was further analyzed. The evolution law of damage quantity, morphology change, and surface quality in the process of MR repairing was studied. Combined with the research results of the MR repairing, a set of perfect repairing process systems was summarized; finally the repairing of small-scale damage of fused silica components cluster was realized.

Based on the existing research, this paper studies the MR removing method as a repairing method to ensure the optical properties and surface quality are maintained, as well as to realize the efficient repairing of small-scale damage.

In the second section, the rule of the MR removing method is studied, and its feasibility and effectiveness are analyzed. The optimal repairing depth and the problems after repairing are summarized based on the damage repairing process.

In the third section, we carried out research on the optimization of the repairing process. By analyzing and solving the problems in the MR repairing process, a better MR repairing process is proposed. The main process includes three steps: 1. MR repairing of 4 μm; 2. computer controlled optical surfacing (CCOS) repairing of 700 nm; and 3. CCOS polishing of 200 nm. The repair effect and surface quality can meet the actual system index.

The fourth section focuses on the experimental verification of the optimized repairing process. The results show that the repairing rate of small-scale damage can be up to 90.4%, and the optical properties and surface quality of the components can basically return to the level before damage.

In general, the MR repairing technique can be used to repair small-scale damage of fused silica. The experimental results verify the effectiveness and feasibility of fused silica component repair. This new repairing method can bring new enlightenment to the existing repairing technology, and is conducive to improve the service life of fused silica high-intensity light components.

## 2. Experiment of MR Repairing Damage

### 2.1. Experimental Parameters

In order to verify the feasibility of repairing small-scale damage of fused silica with the MR removing method and study the evolution law of the number, morphology, and surface roughness of the small-scale damage points, two fused silica samples with size of 50 mm × 50 mm × 10 mm and the material of Heraeus 312 were used. The surface roughness of the component was 0.975 nm, which met the requirements of the actual system.

A pulsed laser with 355 nm wavelength and 7 ns pulse width was used to irradiate one the fused silica elements through three scans to prepare the damaged element, and the other fused silica element was not damaged. The average output energy of the laser was about 80 mJ, the spot area was about 1.5 mm^2^, and the calculated energy density was about 5.4 J/cm^2^ (the actual system is about 5 J/cm^2^).

The KDUPF-700 MRF, which is made by National University of Defense Technology, Changsha, China, was used to remove the surface of damaged and non-destructive components evenly. Cerium oxide was used to remove the wear particles, and hydroxy iron powder was used as soft powder. The specific process parameters are shown in Table 1. After removing 1 μm each time, absolute ethanol was used to wipe the surface of the component. Then HF acid was used to remove the hydrolytic layer, and then it was cleaned and dried. Finally, the number and shape evolution of small-scale damage were measured by an ultra-smooth surface laser scattering defect detector, an ultra-deep hole microscope, and an atomic force microscope. The surface roughness evolution of the experimental component was measured using a white light interferometer.

### 2.2. Evolution of the Number and Morphology of Small-Scale Damage

**Figure 3:**
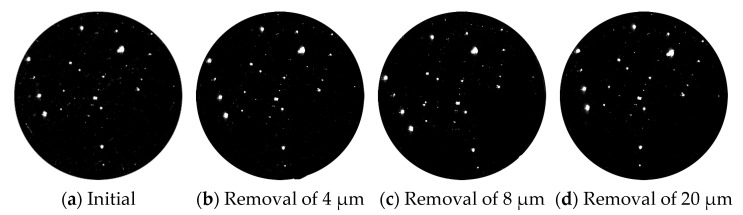
Dark field results of damage with different sizes before and after MR repair.

Using the laser scattering defect detector of super-smooth surface, which is made by ZC Optoelectronic Technologies, Ltd., Hefei, China, to measure the evolution of damage quantity in the process of the MR repairing, the dark field image was obtained, as shown in Figure 3. Table 2 shows the change of each size of damage points with the MR removal depth after image processing. Based on the comparison of the test results, the number of component damage points after MR repairing was significantly reduced. When the removal depth was up to 20 μm, the total number of damage points was reduced by 80%. The number of damage points with sizes larger than 20 μm were basically unchanged, but the small-scale damage points with sizes smaller than 50 μm were reduced by almost 90%. This showed that this method can effectively remove a large number of small-scale damage points.

**Figure 4:**
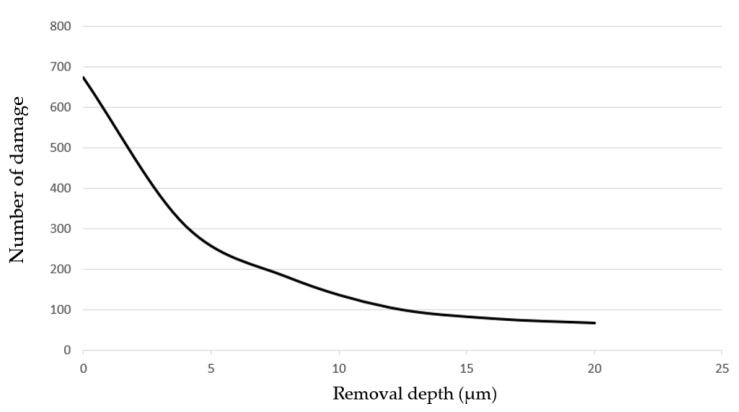
Variation of damage number where damage size was smaller than 50 μm with removal depth.

The curve of damage number with transverse sizes smaller than 50 μm variation with the MR removal depth were drawn, as shown in Figure 4. The results showed that the repairing efficiency was very high at the beginning, but the efficiency gradually reduced as the removal depth increased. When the removal depth was up to 20 μm, the overall removal rate of small damage points met the requirements.

**Figure 5:**
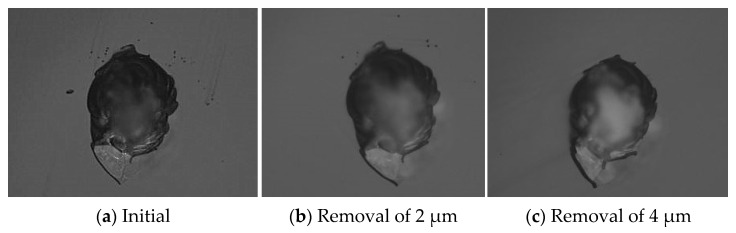
Morphology variation of large-scale damage.

Variations in large-scale damage were also observed using the optical microscope, as shown in Figure 5. The results showed that there were clusters of small-scale damage around areas of large-scale damage before the MR repairing. When the removal depth was up to 2 μm, the number of the small-scale damage points were significantly reduced, but it was not completely repaired. When the removal depth was increased to 4 μm, the small-scale damage was basically repaired. However, it was clearly observed that the trailing phenomenon occurred at the damage point. The trailing phenomenon at the small damage points was completely removed in the MR repairing process, but the trailing phenomenon at the large damage points became serious.

**Figure 6:**
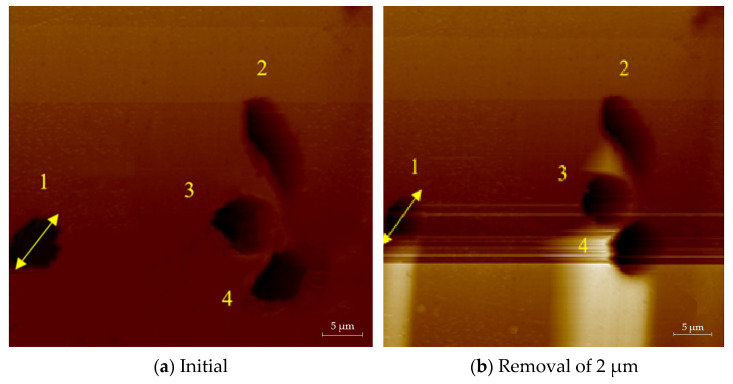
Morphology evolution of small-scale damage before and after MR removal.

**Figure 7:**
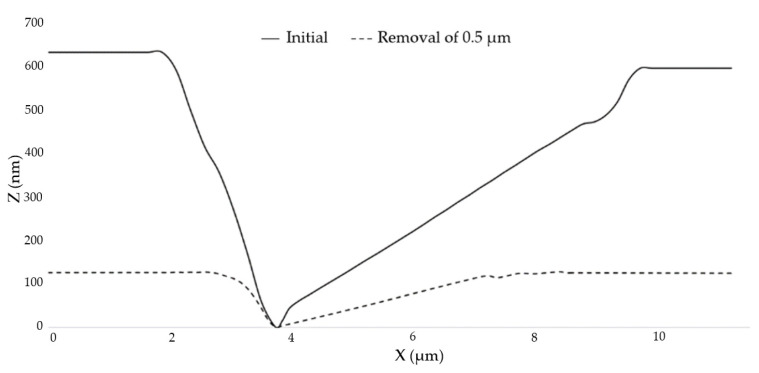
Profile evolution of damage before and after MR removal.

The atomic force microscope was used to randomly select small-scale damage and observe its repairing evolution process, as shown in Figure 6. Figure 6a is the initial image of the small-scale damage on the element. After removing 0.5 μm uniformly with the MR removing method, the measured results are shown in Figure 6b. The transverse size of the damage became smaller and the damage contour became more regular, as observed by comparing the two images. The profile of damage 1 measured along the MR scanning direction is shown in Figure 7. The width of the damage decreased from 7 μm ± 10% to 4.5 μm ± 10%, and the depth decreased from 620 nm to 120 nm. The depth was basically the same as the removal depth of MR, and the contour of the damage opening became smoother. The results showed that the variation of damage depth was consistent with the MR removing depth, and the repairing process did not cause damage to the bottom of the damage. This indicates that the MR removing method can effectively repair small-scale damage.

### 2.3. Evolution of Surface Roughness

**Figure 8:**
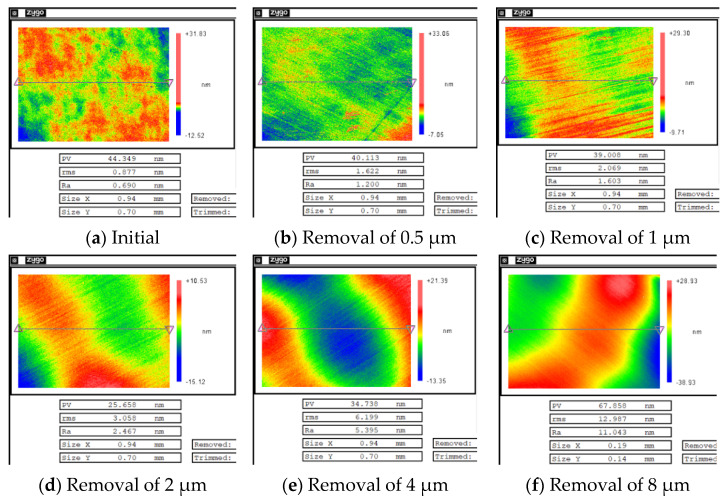
Results of surface roughness of a non-destructive component with different removal depths.

After magnetorheological treatment of the non-destructive component, the white-light interferometers with removal depths of 0.5, 1, 2, 4 and 8 μm were selected. The measurement results are shown in Figure 8. A three-dimensional white light scanning interferometer (NewView 700) made by Zygo Corporation, Connecticut, United States, was used to measure the surface roughness of optical elements. The device is a non-contact instrument for measuring the surface roughness of optical elements. Its field of view is 0.94 mm × 0.70 mm, the longitudinal resolution is 0.1 nm, the transverse resolution is 0.36–9.50 μm, and the measurement repeatability Root Mean Square (RMS) is less than 0.01 nm [22].

**Figure 9:**
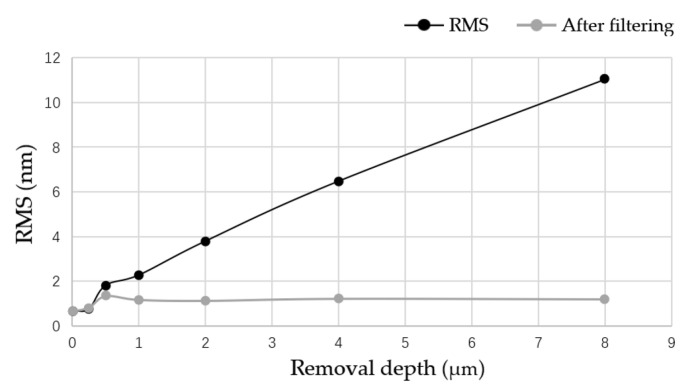
Evolution of surface roughness of a non-destructive component by removal depth.

According to the division of error frequency band by Lawrence Livermore National Laboratory (LLNL), an error with F > 8.33 mm−1 was considered a high-frequency error. In order to study the variation of errors in different frequency bands by the removal depth, mid-frequency errors of F ≤ 8.33 mm^−1^ were filtered out in the measurement, and the variation results of surface roughness with the removal depth before and after filtering were plotted, as shown in Figure 9.

The results show that the surface roughness of the non-destructive component increased linearly with the increase in the removal depth, and the high-frequency error of the components did not change with the removal depth. This indicates that the MR removing method leads to the increase in the mid-frequency and low-frequency error of the component, which are mainly caused by the convolution effect of the MR removing method. In addition, with the increase in the removal depth, the high-frequency error of the non-destructive element was found to first deteriorate and then tended towards stability, which was mainly determined by the initial surface roughness of the element and the characteristics of the removal function.

**Figure 10:**
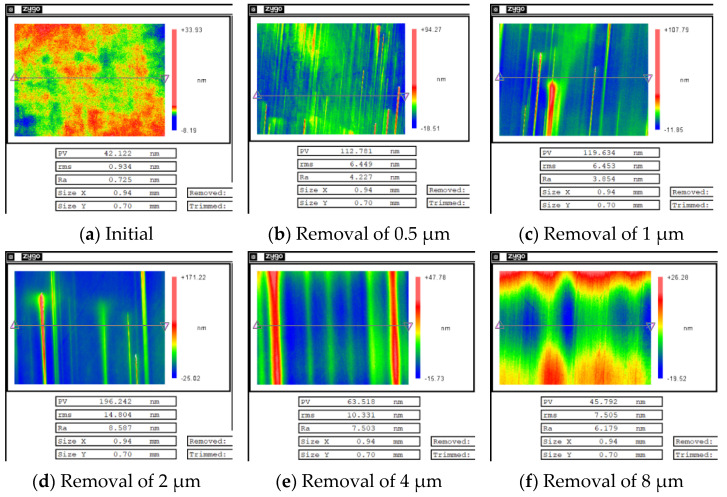
Results of surface roughness of damaged component with different removal depths.

After MR repairing of the damaged component, white-light interferometers with removal depths of 0.5, 1, 2, 4, and 8 μm were selected. The measurement results are shown in Figure 10.

**Figure 11:**
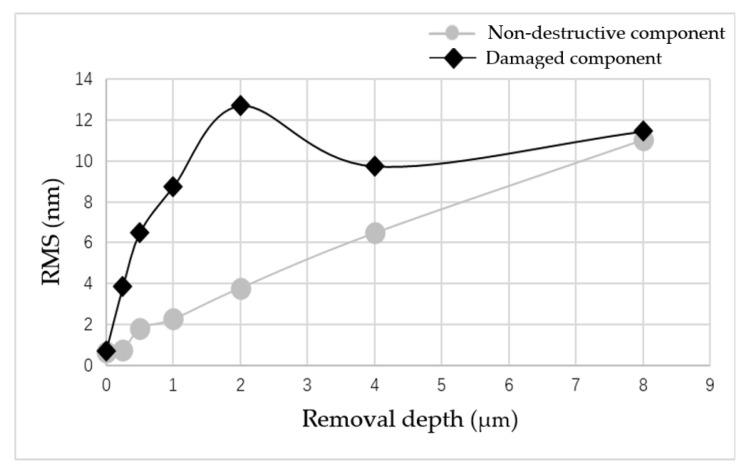
Comparison of surface roughness changes of the two components.

The curves of surface roughness of the non-destructive component and damaged component with different removal depths are plotted together, as shown in Figure 11.

The results showed that when the repairing depth was smaller than 4 μm, the RMS increased rapidly and was is much higher than for the RMS of the non-destructive component. Then, with the increase in removal depth, RMS gradually decreased, and finally it became similar to the RMS of the non-destructive component. From the measurement results in Figure 10, it was found that during the rapid increase in RMS, many non-uniform strip tails were produced on the component surface, which gradually disappeared with the increase in the removal depth. The measurement results showed that in the process of the MR removing method, the existence of damage led to the tailing phenomenon, which was one of the reasons for the rapid deterioration of the surface quality of components. However, with the further increase in the removal depth, the influence of damage on the surface quality was gradually eliminated.

## 3. Repair Process Optimization Strategy

In the second part, the damage law of fused silica repaired by MR was studied, and the feasibility and effectiveness of the MR repairing were analyzed. The results showed that MR repairing can effectively repair small damage points, and the removal rate can be more than 90%. At the same time, it will also lead to two problems: firstly, with the increase in the repairing depth, the surface roughness of component increases, which will not meet engineering needs; secondly, there will exist the tailing phenomenon in the process of repairing damage. In order to solve the series of problems induced by the MR repairing process, computer controlled optical surfacing (CCOS) was used to modify the repaired components, and a perfect combination repair process is achieved by combining with the MR repairing law.

According to the damage law of fused silica repaired by MR, with the increase in the MR repairing depth, the tailing of large-scale damage became deeper, and the surface roughness worsened. In order to eliminate tailing and restore the surface roughness as much as possible, the appropriate process parameters of MR and CCOS were selected.

**Figure 12:**
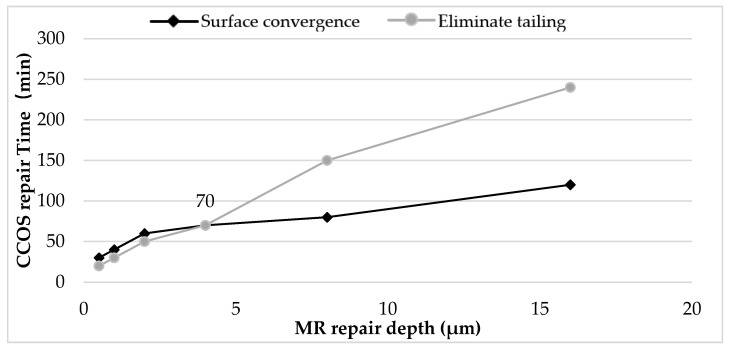
Relationship curves of MR and CCOS process parameters.

This section studies the time required for CCOS to eliminate tailing and restore surface roughness under different MR repairing depths. The results are shown in Figure 12. The black curve in the figure shows the time required for CCOS to recover the surface roughness at different MR repairing depths, and the gray line shows the time required for CCOS to eliminate tailing under different MR repairing depths. The results show that when the MR repairing depth was 4 μm, CCOS repairing needed 70 min to eliminate the tailing and restore the surface roughness. At this time, the revolution speed of CCOS was 150 rotations per minute (rpm) and the diameter of polishing powder was 1.5 μm. Other process parameters are shown in Table 3, and the corresponding depth of 70 min was 700 nm.

**Figure 13:**
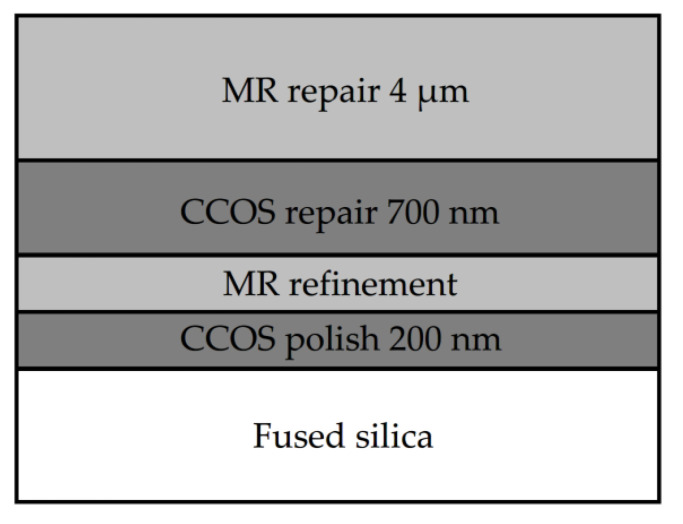
Material removal distribution of each process.

Finally, in order to further improve the surface smoothness and eliminate the sub-surface defects, the combined process parameters were optimized based on the previous process research conclusions. The final repair process is shown in Figure 13. The combination process is as follows: MR repairing of 4 μm; CCOS repairing of 700 nm with revolution speed of 150 rpm and polishing powder diameter of 1.5 μm. After two cycles to achieve the ideal repair effect, MR refinement was carried out. Finally, CCOS repairing of 200 nm with a revolution speed of 0 rpm and polishing powder diameter of 0.5 μm should be used. At this time, the damage repair effect and surface quality of components were able to meet the system index.

## 4. Experimental Verification of Repair Process

**Figure 14:**
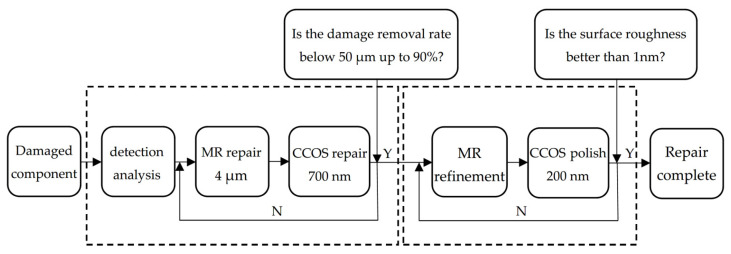
Repair flow chart.

Figure 14 shows the flow chart of the whole repairing process. The repair of small-scale damage of fused silica was divided into three stages: the damage quantity and size distribution detection; the MR repairing of small-scale damage; and surface quality recovery. The specific process steps were as follows: (a) the initial damage detection was carried out on the existing fused silica damage elements and the distribution characteristics including the number and size of the damage was obtained; (b) the MR removing method was used to repair 4 μm, and CCOS with revolution speed of 150 rpm and polishing powder diameter of 1.5 μm was used to repair 700 nm. Two cycles were carried out until the removal rate of small-scale damage was of more than 90%; (c) MRF polishing was used, and then CCOS with a revolution speed of 0 rpm and polishing powder diameter of 0.5 μm was used to polish 200 nm until the surface roughness was better than 1 nm. Finally, the components were cleaned.

**Figure 15:**
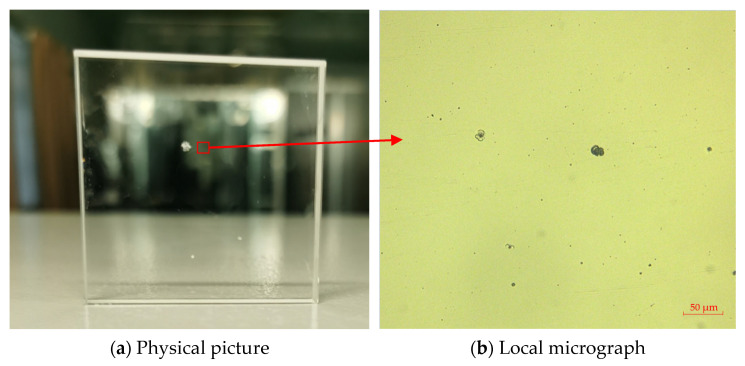
Physical and micrographs of damaged components.

In order to verify the effectiveness of the whole repairing process, the above repairing process was used to repair the fused silica damaged components produced by an actual system. The experimental sample was a fused silica damaged component of 50 mm × 50 mm × 10 mm. Three large-scale damages were directly observed on the surface of the component. The local image in the red frame was observed through the optical microscope, as shown in Figure 15. There were also clusters of small-scale damage on the surface of the components.

**Figure 16:**
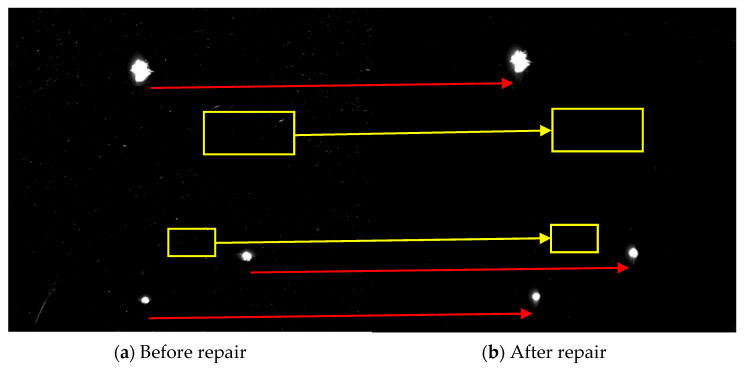
Dark field images of damaged components before and after repairing.

**Figure 17:**
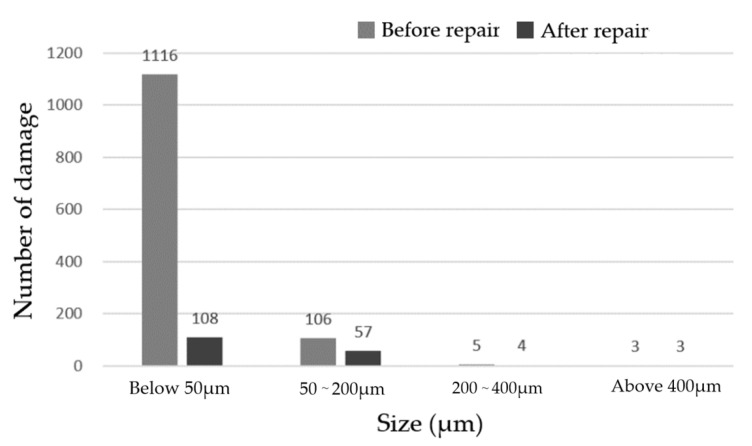
Number of different sizes of damage before and after repairing.

The MR repairing method was used to repair 4 μm, and the CCOS was used to repair 700 nm. After the process was completed, the removal rate of small surface damage was more than 90%, and the removal depth was 23.5 μm. Figure 16 shows the comparison of dark field images before and after the MR repairing. Figure 17 shows the distribution of the number of damage points with different sizes. The results show that after the MR repairing, the number of damage points, especially damage points with sizes smaller than 50 μm, were significantly reduced, with a removal rate of 90.4%.

**Figure 18:**
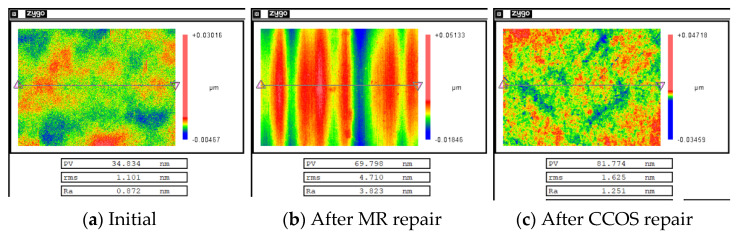
Evolution of surface roughness during the process.

Figure 18 shows the evolution of the surface roughness during the repair process measured by the white light interferometer. The surface roughness of the repaired component was 1.625 nm, which did not meet the system index.

**Figure 19:**
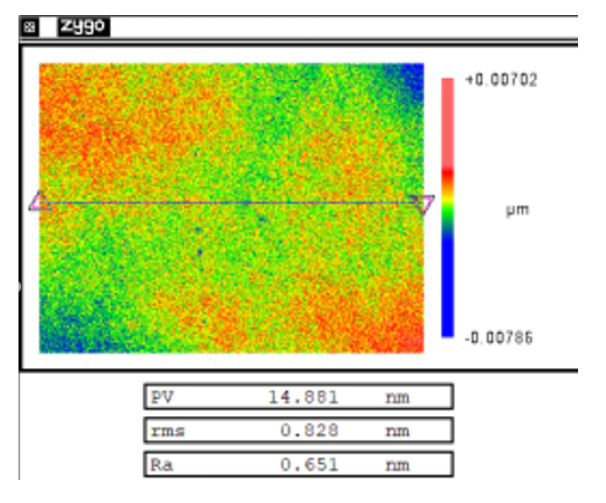
Result of surface roughness after refinement.

The surface roughness of the components was restored by combining MR refinement and CCOS polishing. Figure 19 shows the measurement result after the surface quality was restored. At this time, the surface roughness was 0.828 nm, which met the requirement that the surface roughness is better than 1 nm.

## 5. Conclusions

The research on the MR repairing fused silica damaged components was carried out, and the rapid repair of fused silica small-scale damage was successfully realized. The repairing rate of small-scale damage was more than 90%, and the optical properties and surface quality was able to reach the system index.

Firstly, it was found that the size and quantity distribution of the damage were key factors affecting the repairing process. The number of cluster small-scale damage, the morphology and depth of single small-scale damage, and the evolution law of fused silica component surface quality were studied. The feasibility of the MR removing method to remove the damage points was verified. The problems after repairing were analyzed, the causes of the problems were explored, and the solutions were summarized.

Secondly, the optimization of the restoration process was studied. By selecting the appropriate process parameters for MR and CCOS, the surface roughness was able to be restored as much as possible, while eliminating the tailing caused by the MR removing method. Finally, the optimized repairing process parameters were obtained: MR repairing for 4 μm, CCOS repairing for 700 nm with revolution speed of 150 rpm and polishing powder diameter of 1.5 μm. After two process cycles, MR refinement was carried out. Finally, CCOS with a revolution speed of 0 rpm and a polishing powder diameter of 0.5 μm was used to polish 200 nm. The damage repair effect and surface quality of the components met the actual system index.

Finally, a repairing process validation experiment was carried out. The repairing results showed that the repair rate of small-scale damage can be up to 90.4%, and the optical properties and surface quality of the components can be restored to the level before damage. The experimental results verify the effectiveness and feasibility of MR repairing.

In sum, the MR removing method can be used to repair small-scale damage of fused silica components. This new repairing method can complement existing repair technology and is conducive to improving the service life of fused silica optical components.

## Figures and Tables

**Table 1:** Parameters of the MR process.

Item	Level
Wheel speed (r/min)	280
Flow rate (L/min)	120
Current (A)	8
Ribbon penetration depth (mm)	0.25

**Table 2:** Quantity statistics of damage before and after MR repair.

	Removal Depth	0–50	50–200	200–400	Larger Than 400
Size (μm)					
0 μm		673	86	21	24
4 μm		307	67	21	24
8 μm		180	55	21	24
12 μm		105	49	20	24
16 μm		78	46	20	24
20 μm		67	44	20	24

**Table 3:** Parameters of the CCOS process.

Item	Level
Polishing powder material	Cerium dioxide
Disc material	asphalt
Disc diameter (mm)	25
Rotation speed (rpm)	155
Eccentricity (mm)	5
Polishing pressure (KPa)	50

## Data Availability

The data presented in this study are available on request from the corresponding author. The data are not publicly available due to the data also forms part of an ongoing study.
